# Analysis of two different luteal phase support regimes and evaluation of *in vitro* fertilization-intra cytoplasmic sperm injection outcomes

**DOI:** 10.4274/tjod.73603

**Published:** 2019-01-09

**Authors:** Nafiye Karakaş Yılmaz, Mustafa Kara, Necati Hançerlioğulları, Selçuk Erkılınç, Buğra Coşkun, Ayla Sargın, Salim Erkaya

**Affiliations:** 1 University of Health Sciences, Zekai Tahir Burak Women’s Health Training and Research Hospital, Clinic of Obstetrics and Gynecology, Ankara, Turkey; 2 Bozok University Faculty of Medicine, Department of Obstetrics and Gynecology, Yozgat, Turkey

**Keywords:** Luteal phase support, gonadotropin-releasing hormone agonist, infertility, in vitro fertilizationn-intra cytoplasmic sperm injection

## Abstract

**Objective:**

To evaluate clinical pregnancy rates, miscarriage rates, ongoing pregnancy rates, and in vitro fertilization-intra cytoplasmic sperm injection outcomes of gonadotropin releasing hormone agonist (GnRHa) administration compared with human chorionic gonadotropin (hCG) application for luteal phase support.

**Materials and Methods:**

A total of 456 patients were included in the study. The patients were divided into two groups according to luteal phase support type: in group 1 (n=158), single-dose triptorelin acetate 0.1 mg was given on the sixth day after the oocyte pick-up (OPU). In group 2 (n=298), hCG 1500 IU was given on day 4, 7 and 10 after the OPU.

**Results:**

Both groups were homogeneous in relation with age and antral follicle count. The number of stimulation days and endometrial thickness on hCG day (mm) were found to be significantly higher in group 2 than in group 1 (p<0.001). The clinical pregnancy rate was slightly higher in the GnRHa group, but this difference was not statistically significant.

**Conclusion:**

Although there was no statistically significant difference between the two groups, luteal phase support with single-dose GnRHa might be as efficient as three doses of hCG. Large prospective, randomized-controlled studies are required comparing GnRHa and hCG for luteal phase support.

**PRECIS:** There was no gap with GnRHa for luteal support.

## Introduction

*In vitro *fertilization (IVF) - intra cytoplasmic sperm injection (ICSI) has been used worldwide for more than two decades and embryo implantation is a major component of this procedure. Optimization of endometrial receptivity is essential for a successful implantation^(1)^. Supraphysiologic estradiol (E_2_) levels due to controlled ovarian hyperstimulation (COH) lead to a decrease in luteinizing hormone (LH) levels. The corpus luteum may not be functional in the absence of LH. The occurrence and maintenance of pregnancy necessitates adequate secretion of progesterone from the corpus luteum^(2,3)^. Dysfunction of the corpus luteum results with low progesterone levels. Therefore, a progesterone supplement is administered during the luteal phase to achieve optimal endometrial receptivity^(4)^. There are many protocols of luteal support in assisted reproductive technology (ART) cycles. Luteal phase support with progesterone is a standard approach for ART cycles^(5)^. Progesterone can be used via oral, intramuscular, and transvaginal routes. However, there is still debate about the starting time and continuation. Transvaginal progesterone is commonly used for luteal phase support. Progesterone administration is initiated on the oocyte pick-up (OPU) day and continued for 12 days, until the serum beta human chorionic gonadotropin (hCG) measurement day. However, there are conflicting results regarding the dose, route of administration (oral, subcutaneous, transvaginal), duration (until the ultrasound demonstration of heartbeat in an intrauterine gestational sac, until 10 weeks of gestation, until 12 weeks of gestation), and formulations such as synthetic or micronized types of progesterone. Although some studies indicated that transvaginal progesterone use was efficient for luteal phase support, Vaisbuch et al.^(6)^ reported that further studies were necessary for this subject. Pritts et al.^(7)^ reported that the addition of E_2 _to progesterone could be more effective on IVF-ICSI outcomes. A Cochrane review was reported by Daya et al.^(8)^ regarding luteal phase support in ART cycles. The authors concluded that luteal phase support with hCG or progesterone after ART was associated with an increased clinical pregnancy rate [odds ratio (OR) 1.34, 95% confidence interval (CI): 1.01-1.79]. They found that luteal phase support with hCG had grater Ovarian hyperstimulation syndrome (OHSS) risk than progesterone (OR 3.06, 95% CI: 1.59-5.86). Luteal phase support with gonadotropin releasing hormone agonist (GnRHa) was first described by Tesarik et al^(9)^. They found that GnRHa might have a direct effect on the embryo. Other theories about GnRHa are its flare-up action and direct effect on endometrium^(10)^. Although there are many studies about the use of GnRHa as a supporter of luteal phase, the exact mechanism remains controversial. Besides, the results of these studies are conflicting^(11,12)^. Fusi et al.^(13)^ administered GnRHa for luteal support in women at high risk for OHSS undergoing IVF. They concluded that luteal support with GnRHa could be used as the first choice in patients at high risk for OHSS. Engman et al.^(14)^ found that a GnRHa trigger was effective in the prevention of OHSS during IVF treatment. A current Cochrane review was reported by van der Linden et al.^(15)^ about luteal phase support in subfertile women undergoing assisted reproduction. They reported that the addition of GnRHa to progesterone was associated with an improvement in pregnancy outcomes. For this reason, we aimed to compare two different luteal phase support regimes, GnRHa and hCG, and to assess IVF-ICSI outcomes.

## Materials and Methods

### Study design

This study was designed as a prospective cohort trial. In total, 456 women aged between 25 and 38 years were included in the study. The data of the patients were collected from patients who presented to the IVF unit of Ankara Zekai Tahir Burak Women’s Health Training and Research Hospital. The study protocol and ethical consent was approved by the local ethics’ committee. The patients were divided into two groups according to luteal phase support type. Group 1 contained patients who received single-dose triptorelin acetate 0.1 mg. Group 1 included women who underwent IVF-ICSI one year prior to the beginning of the study. Accordingly, this line of the study was retrospective. Group 2 included patients who received hCG 1500 IU, which was given on day 4, 7, and 10 after the OPU. Group 2 joined the study six months after the onset of the study; therefore, this line of the study was prospective. Luteal phase support was given with hCG or GnRHa for 1 year and with GnRHa for six months, in addition to transvaginal progesterone. Subjects who had undergone frozen-thawed embryo transfer and those with male factor infertility were not included in the study. Normoresponder patients were included in the study. Exclusion criteria were having follicle-stimulating hormone (FSH) >15 IU/L, anti-mullerian hormone level <1.0 ng/mL, and an antral follicle count (AFC) <4 on the second day of menstruation. First fresh cycles of all patients were included in the study. Single embryo transfer was performed in all subjects according to legal requirements.

### Gonadotropin stimulation for assisted reproductive technique, oocyte retrieval, and sample collection

All patients were treated with an antagonist protocol, and an hCG trigger was used for final maturation. Flexible daily GnRH antagonist protocol was preferred to induce pituitary down regulation (Cetrotide^®^ 0.25 mcg, Merck-Serono, Switzerland). One hundred fifty-two hundreds twenty five IU daily rec-FSH (Gonal-F^®^, Merck-Serono, Switzerland) and/or human menopausal gonadotropin (Menogon^®^, Ferring, Germany) were started on day 3 of the cycle and continued for the first 3 days of stimulation, after which daily dosing was determined individually. The GnRH antagonist was started when the leading follicle reached a diameter of 12-14 mm. Serial E_2_ levels and two-dimensional follicle measurements using transvaginal ultrasound imaging (Logic 200 Pro^®^, General Electric, Korea) were performed until at least two dominant follicles reached dimensions of 18 mm or greater in diameter. Human chorionic gonadotropin (Pregnyl^®^ 10.000 U I.M., Organon, Netherland) was administered, followed by transvaginal oocyte retrieval 36 h later. ICSI was performed in all patients. Single embryo transfer was used because of legacy. Embryos were classified according to the number of blastomeres, percentage of fragmentation, and blastomere appearences on the first, third, and fifth days. All transfers were made using Rocket ThinWall Transfer Sets (Rocket Medical, Hingham, MA, USA). The patients were allocated into two groups according to luteal phase support type: in group 1 (n=158), single-dose GnRHa triptorelin acetate (Decapeptyl^®^ Ferring, Germany) 0.1 mg was given on the sixth day after the OPU. In group 2 (n=298), hCG 1500 IU was given on days 4, 7, and 10 after the OPU. All women were administered vaginal progesterone (Crinone 8% vaginal gel^®^, Merck-Serono, Switzerland) 90 mg daily starting on the day of oocyte retrieval and lasting for 12 days (until the day of serum b hCG measurement). If pregnancy occurred, progesterone was given until 12 weeks of gestation. Clinical pregnancy was diagnosed through the ultrasound demonstration of heartbeat in an intrauterine gestational sac. Miscarriage rates and ongoing pregnancy rates were calculated.

### Statistical Analysis

Statistical analysis was performed using the SPSS Ver. 15.00 (SPSS Inc., Chicago) statistics software package. Data normality was assessed using the Kolmogorov-Smirnov test. Statistical comparisons between groups were performed using the Mann-Whitney U (for unrelated samples) and Wilcoxon (for related samples) tests. The chi-square test was used for categorical variables and an independent Sample t-test was used for continuous variables that were normally distributed. P<0.05 was considered significant.

## Results

In total, 456 women were included into the study. Single-dose triptorelin acetate was administered to 158 women. Three doses hCG were given to 298 women. The characteristics of the participants are shown in Table 1. Both groups were homogeneous in relation to patients’ age, duration of infertility, basal FSH levels, basal E_2_ levels, and AFC. There were no statistical differences in terms of these parameters. There were no differences between the groups regarding total gonadotropin dose, oocyte number, and metaphase 2 oocyte number (Table 2). Although the clinical pregnancy rate was slightly higher in the GnRHa group, the difference was not statistically significant (p=0.49). Miscarriage rates and live birth rates were not statistically significant between the two groups (p=0.12 and p=0.88, respectively). No systemic adverse effects were observed and no severe OHSS occurred.

## Discussion

In this cross-sectional study, we aimed to compare the efficacy of two different luteal phase support regimes (triptorelin acetate and hCG) and to evaluate IVF-ICSI outcomes. The results of our study demonstrated that administration of triptorelin acetate (GnRHa) might be as efficient as hCG as an agent for luteal phase support. Our results were consistent with previous studies^(8,12)^. COH with GnRH agonists or antagonists has been used to prevent premature luteinization in ART cycles. However, these agents may inhibit the function of the corpus luteum by decreasing LH levels^(16)^. Therefore, luteal phase support in ART cycles has been taken into consideration to avoid this inhibition. Also, these drugs could have direct effects on the endometrium and embryo. According to the world data, those applied drugs were different from others for luteal support^(6)^.

Tesarik et al.^(9)^ first reported that supplementation of single-dose 0.1 mg triptorelin could enhance IVF-ICSI outcomes. The effect of GnRHa given during luteal phase on clinical pregnancy rates is still unclear. On the other hand, GnRHa receptors have been found on the embryo and endometrium. It is speculated that single-dose GnRHa administered during the luteal phase could enhance implantation because it decreases rates of abortion and OHSS, and increases multiple pregnancy rates^(17,18)^. We had no cases of multiple pregnancy because single embryo transfer was performed in all subjects. Pirard et al.^(19)^ suggested that, the addition of a GnRHa (buserelin) during the luteal phase of IVF cycles might be effective for luteal phase support. On the contrary, Ata et al.^(20)^ found that single-dose 0.1 mg triptorelin administration 6 days after ICSI did not increase ongoing pregnancy rates. In our study, miscarriage rates and live birth rates were not statistically significantly different between the two groups (p=0.12 and p=0.88, respectively). The primary endpoint of the present study was to compare the live birth rate. These results were our preliminary findings. The limitations of our study were the restricted number of patients and its retrospective nature. Van der Linden et al.^(21)^ reported that progesterone enhanced the implantation rate, pregnancy rate, and live birth rate. Even though the route of administration did not affect the results, vaginal and intramuscular progesterone were found to be more efficient than the other routes^(21)^. The above-mentioned studies suggested that vaginal progesterone was sufficient for luteal phase support. However, the ideal method remains unclear and the preferences for use are different.

A recent meta-analysis by Kyrou et al.^(22)^ reported on the influence of luteal single-dose GnRHa on IVF-ICSI outcomes. They performed a computerized literature search up until December 2010. From the 38 studies yielded, 6 randomized controlled trials (RCT) were analyzed. The authors concluded that the addition of GnRHa during the luteal phase increased live birth rates. Another recent meta-analysis by Martins et al.^(23)^ documented the effect of GnRHa during the luteal phase for women undergoing ARTs. They found that the use of GnRHa as a luteal phase supporter was still controversial because there was no evidence with respect to adverse perinatal outcomes and congenital malformations. Our results indicate that there was no difference between the GnRHa group and the hCG group in terms of pregnancy rates. The purpose of our study was to compare hCG versus GnRHa protocols as luteal phase support regimes and to evaluate IVF-ICSI outcomes.

## Conclusion

In conclusion, there were no differences in live birth rates between luteal GnRHa and hCG administration in addition to transvaginal progesterone. However, large RCTs are required to determine the effect of luteal phase support with GnRHa on IVF-ICSI outcomes.

## Figures and Tables

**Table 1:**
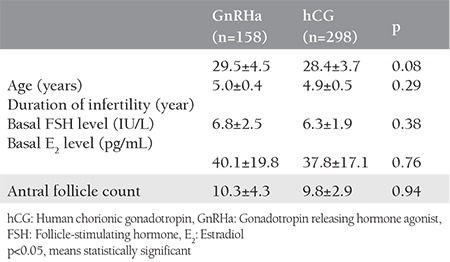
Characteristics of the patients

**Table 2:**
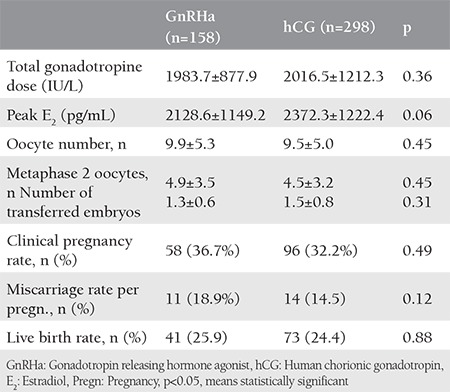
Comparison of *in vitro* fertilization-intracytoplasmic sperm injection outcome according to the luteal phase support
